# Considering Envy and Rivalry within the nomological network of pathological narcissism: an empirical study

**DOI:** 10.1192/j.eurpsy.2022.1715

**Published:** 2022-09-01

**Authors:** F. Faccini, G. Rogier, R. Cavalli, P. Velotti

**Affiliations:** 1 University of Rome Sapienza, Dynamic And Clinical Psychology And Health, Rome, Italy; 2 University of Genoa, Department Of Educational Sciences, Genoa, Italy

**Keywords:** narcissism, envy, Rivalry

## Abstract

**Introduction:**

Few is known regarding the intervening variables between pathological narcissism and sadism personality. Specifically, envy is a psychoanalytical construct that appears especially promising in illuminating such relationships.

**Objectives:**

To extend the knowledge regarding the nomological network of pathological narcissism.

**Methods:**

We administered to a sample of Italian adults a battery of self-report questionnaires including the Italian version of the Benign and Malicious Envy Scale, the Assessment of Narcissistic Personality, The Narcissistic Admiration and Rivalry Questionnaire and the Pathological Narcissism Inventory.

**Results:**

First, the Italian version of the Benign and Malicious Envy Scale showed good fit indexes confirming the original factorial structure as well as configural invariance. We found that only the grandiosity facet of the Pathological Narcissism Inventory, the Rivalry subscale of the Narcissistic Admiration and Rivalry Questionnaire and the Malicious subscale of the Benign and Malicious Envy Scale positively and significantly predicted Assessment of Narcissistic Personality scores. Moreover, throughout a structural equation modeling approach, the hypothesis that rivalry and malicious envy both mediate the relationship between grandiosity and sadism was empirically supported.

**Conclusions:**

The use of the Benign and Malicious Envy Scale resulted to be promising in the investigation of the nomological network of pathological narcissism. Limitations and future directions are discussed.

**Disclosure:**

No significant relationships.

